# Nanohydroxyapatite Application to Osteoporosis Management

**DOI:** 10.1155/2013/679025

**Published:** 2013-10-28

**Authors:** Zairin Noor

**Affiliations:** Department of Orthopaedics and Traumatology, Ulin General Hospital, Faculty of Medicine, University of Lambung Mangkurat, 70232 Banjarmasin, South Kalimantan, Indonesia

## Abstract

Hydroxyapatite is chemically related to the inorganic component of bone matrix as a complex structure with the formula of Ca_10_(OH)_2_(PO_4_)_6_. Previous studies have reported the application of microsized hydroxyapatite to bone regeneration, but the result is not satisfied. The limitation comes from the size of hydroxyapatite. In addition, the duration of treatment is very long. The advantages of hydroxyapatite nanocrystal are the osteoconduction, bioresorption, and contact in close distance. Crystal in osteoporotic bone is calcium phosphate hydroxide with the chemical formula of Ca_10_(OH)_2_(PO_4_)_6_. Crystal of normal bone is *sodium calcium hydrogen carbonate phosphate hydrate* with the chemical formula of Ca_8_H_2_(PO_4_)_6_ *·*H_2_O–NaHCO_3_–H_2_O. The recent development is applying nanobiology approach to hydroxyapatite. This is based on the concept that the mineral atoms arranged in a crystal structure of hydroxyapatite can be substituted or incorporated by the other mineral atoms. In conclusion, the basic elements of hydroxyapatite crystals, composed of atomic minerals in a certain geometric pattern, and their relationship to the bone cell biological activity have opened opportunities for hydroxyapatite crystals supplement application on osteoporosis. Understanding of the characteristics of bone hydroxyapatite crystals as well as the behavior of mineral atom in the substitution will have a better impact on the management of osteoporosis.

## 1. Background

Bone is an organic-inorganic ceramic composite containing well-structured collagen fibrils, nanocrystalline, and rod-like inorganic material with length of 25–50 nm. Sequence of bone structure is formed from seven levels of hierarchy and reflects the material and mechanical properties of each component. Hydroxyapatite is chemically related to inorganic component of bone matrix as a complex structure with formula Ca_10_(OH)_2_(PO_4_)_6_. Similarity of chemical compound of the hydroxyapatite to the bone has triggered intensive researches on the use of synthetic hydroxyapatite as bone substitution and/or bone repositioning for biomedical applications [1].

Intrabone defect is a challenge for clinicians. This defect only requires flap surgery or is associated with other techniques. Bone substitution is necessary to repair segmental defects caused by the removal of infected tissues or bone tumor. Bone substitution mostly required in the particular case is autologous bone. However, autograft is not always available and may cause morbidity in donor site. Allograft may be an alternative in some cases, but there is the possibility of an immune response and disease transmission (HIV and hepatitis B) to the recipient. Bone substitute graft attracts attention among experts due to the advantages that go beyond autograft and allograft. Biomineral morphogenesis is a specific strategy in the development of architectural construction of chemical compounds in microsize and nanocrystal [2]. 

Clinically, osteoporosis is identified through nontraumatic/minimal fracture in the vertebra, hip, proximal humerus, and femur fracture. Osteoporosis is a disorder specifically found in elderly men and women [3–6]. Increased economy and aging population will increase the frequency of osteoporosis so that it becomes an essential health issue [5, 7]. 

This paper will discuss the development of a synthetic material as a bone substitute graft and the potentials of hydroxyapatite microcrystals and nanocrystals, as well as hydroxyapatite nanocrystal applications as a method of filling bone defect in osteoporosis. 

## 2. Synthetic Materials

There are a variety of synthetic materials used in bone substitute graft, including metal, tantalum, titanium, iron, magnesium; polymers such as polylactide, polyglycolide, polyurethane, or polycaprolactone; and ceramics such as silica-based glass, calcium sulfate hemihydrate (CSH or plaster of Paris) and dihydrate (CSD or gypsum), and calcium phosphate. Of these materials, calcium phosphate-based materials are very attractive. Based on its similarity to the composition of the bone structure (about 60% is calcium phosphate), the material was considered for the first time, since a century ago, and has been widely studied as a bone substitution for 40 years [8].

## 3. Bone Grafts

Most of the bone substitute grafts used are granules (diameter between 0.1 to 5 mm) or porous blocks (or sponges). Some formulas may harden after implantation *in situ* or injection. Formulae with such properties, among others, are calcium sulfate hemihydrate (plaster of Paris) and phosphate calcium cement. Reaction setting of this material was initiated by mixing the powder with the liquid solution. Chemically, hardening occurs due to the success of dissolution and precipitation reactions. Mechanically, hardening is caused by entanglement and intergrowth of crystals [8]. 

Possible injection of bone substitute graft has extended its application, for example, the treatment of bone fractures in minimally invasive surgery. In addition to pasta, porous blocks, and pasta that can be hardened, bone substitute graft can also form a paste that cannot be hardened (= putty). This material is a combination of the granules and the “glue” as a highly viscous hydrogel [8]. 

## 4. Hydroxyapatite Potentials

Nowadays, hydroxyapatite is widely used in biomedical applications, including matrices to control drug release and material engineering of bone tissues. Based on the chemical similarities between the hydroxyapatite and inorganic component of bone matrix, synthetic hydroxyapatite has a strong affinity to host hard tissues. Chemical bond between the host and hydroxyapatite causes this material to become clinically a beneficial application, which is better than allograft or metal implants. The main advantages of hydroxyapatite are biocompatibility, low *in situ* biodegradation, and good osteoconduction. In addition, hydroxyapatite also has good biocompatibility to the soft tissues, such as skin and muscles. Synthetic hydroxyapatite is currently widely used for hard tissue repair. Hydroxyapatite is commonly used in bone repair as bone addition, coating implants and bone fillers [1].

## 5. Hydroxyapatite Microcrystals

Calcium hydroxyapatite microcrystal is a natural extract of bone calcium. Calcium hydroxyapatite microcrystal contains a number of minerals in physiological proportions along with other bone organic minerals. Evidence suggesting that calcium hydroxyapatite microcrystal is better absorbed than calcium supplements has triggered a variety of clinical applications. Oral administration of calcium hydroxyapatite microcrystals can accelerate fracture healing and repair and even prevent osteoporosis [9].

## 6. Hydroxyapatite Nanocrystal 

Recent development in biomaterial research focuses on the limitations of calcium phosphate ceramics and improved bioreactivity through the use of nanotechnology. Bone graft of synthetic hydroxyapatite nanocrystal has been introduced in bone defect repair procedure. The advantages of hydroxyapatite nanocrystal are osteoconduction, bioresorption, and contact in close distance. Typical description of materials with nanostructures is a very high number of molecules on the surface of the material. When hydroxyapatite nanocrystal is used as a bone substitute graft, rapid healing of critical size defect has been demonstrated in animal experiments and applications in humans. Hydroxyapatite nanocrystals will be bound to the bone and stimulate bone healing through the stimulation of osteoblast activity. Nonetheless, hydroxyapatite nanocrystal is difficult to form specific formula needed for bone repair and implantation. This is due to the intrinsic hardness, fragility, and lack of the flexibility, thereby restricting the use of a load-bearing implant material. Therefore, hydroxyapatite nanocrystal is often combined with various polymers to produce osteoconductive biocomposite material in the field of orthopedic surgery [1, 10].

## 7. Hydroxyapatite Crystals in Osteoporosis

**Figure 1:**
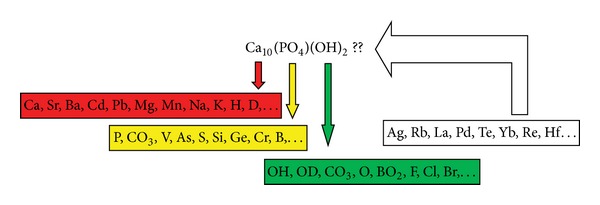
Some compositional possibilities can match the apatite structure. This is the phenomenon that provides a high compositional variation as a reference to the nonstoichiometric character. Besides, there are several atomic minerals which have unknown effect on hydroxyapatite crystals [12–15].

Osteoporosis is a disorder that causes a decrease in bone mass that is normally mineralized due to an imbalance between osteoclast and osteoblast activities [3–5]. Osteoporosis is an amorphous process due to the irregular degenerative process, which is difficult to characterize, within a wide range of bone mineralization. Change in bone mineralization is strongly influenced by the nature of atoms capable of performing a substitution to form a composite as seen in Figure 1 [6, 7].

Solid material is described as amorphous and crystalline material. An amorphous material when its atoms are randomly arranged will be similar to the iron atoms in the liquid. Crystalline materials where the atoms are arranged in a regular pattern with the smallest element in a three-dimensional replication will form crystals [11]. Bone crystals are extremely small, with a mean length of 50 nm (within range of 20–150 nm), an average width of 25 nm (in range of 10–80 nm), and a thickness of only 2–5 nm. Apatite phase contains 4–8% carbonate by weight, referred to as dahlite. Mineral composition will vary with age and is always subject to calcium deficiency where carbonate and phosphate ions are in the crystal lattice. Formula Ca_8.3_(PO_4_)_4.3_(CO)_3*x*_(HPO4)_*y*_(OH)_0.3_ reflects the average composition of the bone, where *y* decreases and *x* increases with age, whereas the addition *x* + *y* will be constant and equal to 1.7. Mineral crystal growth occurs under specific orientation in which the *c-*axis of the crystal is approximately parallel to the length axis of the collagen fiber where the crystal deposition takes place. Electron microscopic technique is used to obtain this information [12].

**Figure 2:**
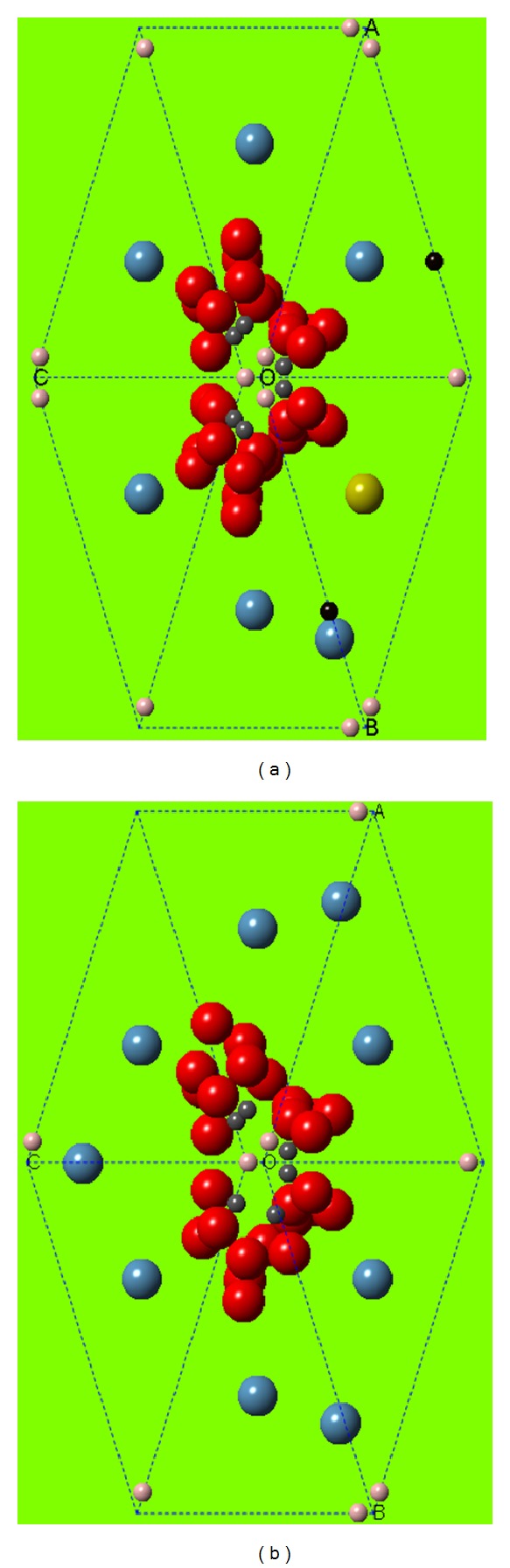
Crystal structure of normal bone (a) and osteoporotic bone (b). Normal crystal structure of the sodium calcium hydrogen carbonate phosphate hydrate with chemical formula Ca_8_H_2_(PO_4_)_6_·H_2_O–NaHCO_3_–H_2_O. Crystal structure of osteoporotic bone is the calcium phosphate hydroxide with chemical formula Ca_10_(OH)_2_(PO_4_)_6_. The difference lies in the number of calcium atoms (local hypermineralization) and the presence of H_2_O–NaHCO_3_–H_2_O (substitution/incorporation) [14].

XRD characterization results with the nanopowder production method using *High Energy Milling* then simulated using Crystal Maker Software as shown in Figure 2, shows that the peak found is the peak of hydroxyapatite crystals, and no other crystalline phases were detected. Crystal in osteoporotic bone is calcium phosphate hydroxide with chemical formula of Ca_10_(OH)_2_(PO_4_)_6_. Crystal of normal bone is sodium calcium hydrogen carbonate phosphate hydrate with chemical formula of Ca_8_H_2_(PO_4_)_6_·H_2_O–NaHCO_3_–H_2_O [14]. The results of search and match test of both normal bone and osteoporotic bone samples show crystalline phase with hexagonal structure. Atom density in osteoporotic bone is lower (0.0841 atom/Å) compared with normal bone (0.0994 atom/Å). Osteoporotic bone crystal size is smaller than normal bone. Furthermore, crystallinity of osteoporotic bone is smaller than normal bone [14]. 

Crystal size in osteoporotic bone is smaller than that in normal bone. This is presumably due to the irregular atomic arrangement in the osteoporotic bone crystal in which crystallographic parameters in osteoporotic bone are not known, while in normal bone, crystallographic parameters are known well in the form of hexagonal crystal system. Moreover, osteoporotic bone crystal showed a lower crystallinity than normal bone. This indicates that the osteoporotic bone crystal has irregular atomic arrangement or amorphous arrangement. For normal bone, the arrangement of atoms in a hexagonal structure has regularity. Besides the atom density of osteoporotic bone crystals is smaller than that of normal bone.

Hexagonal crystal structure has a length or crystal lattice parameter *a* = *b* ≠ *c*, while the angle *α* = *β* = 90° and angle *γ* = 120°. This finding is consistent with a study by Sastry et al. [16], suggesting that the crystallinity of osteoporotic bone decreased. 

## 8. Potential of Hydroxyapatite in Osteoporosis

Ossein microcrystalline hydroxyapatite is a combination of organic and inorganic components. Inorganic components will provide calcium and phosphorus in the physiological ratio 2 : 1. The organic components include collagen and noncollagen proteins with various growth factors and bone specific proteins, such as insulin-like growth factors I and II, transforming growth factor (TGF-*β*), and osteocalcin. As an alternative to calcium, ossein hydroxyapatite compound is a complex protein mineral that has higher osteogenic effect when compared with mineral compounds or calcium supplement administered orally. Ossein hydroxyapatite provides benefits in the treatment and prevention of osteopenia and osteoporosis in women, including primary and secondary osteoporosis. Various studies suggest that ossein hydroxyapatite is more effective than calcium carbonate in reducing bone loss in postmenopausal women and in preventing bone loss as well. 

A study by Castelo-Branco et al. [17] proved that the administration of ossein hydroxyapatite at a dose of 3.32 gram/day compared to calcium carbonate at 2.5 mg/day for 12 and 24 concluded that no change in bone mass is found to be related to the baseline condition in the administration of ossein hydroxyapatite, while the administration of calcium carbonate found a significant decrease in bone mass in the second year. The study by Pelayo et al. [18] stated that the combination of ossein hydroxyapatite and raloxifene was more effective than a combination of ossein hydroxyapatite and calcium carbonate in controlling bone loss among postmenopausal women. A study by Albertazzi et al. [19] that compared ossein hydroxyapatite with tricalcium phosphate at a dose of 500 mg/kg body weight in the prevention of bone loss found that, in the third and sixth months, ossein hydroxyapatite and tricalcium phosphate reduced bone formation markers significantly compared with placebo. In the sixth month, tricalcium phosphate reduced osteocalcin by 9.9% and ossein hydroxyapatite by 12.3%. In addition, they found a decrease in propeptide of type 1 procollagen (PINP) of 5.3% (tricalcium phosphate) and 6.3% (ossein hydroxyapatite). Alkaline phosphatase also decreased by 4.3% (tricalcium phosphate) and 6.7% (ossein hydroxyapatite). Effects on bone resorption markers and bone mineral density did not differ significantly (*P* > 0.05). 

In randomized, open-label, parallel-group, controlled, and prospective study, we compare the effects of OHC (treatment group) and calcium carbonate (control group) on bone metabolism (followed up for a maximum of 3 years). Subjects were women aged >65 years with densitometric osteoporosis of the lumbar spine or femoral neck. The treatment group received open-label OHC (osteoporosis) at a dose of two 830 mg tablets every 12 hours (712 mg elemental calcium per day). The control group received open-label calcium carbonate at a dose of 500 mg of elemental calcium every 12 hours (1000 mg elemental calcium per day). Both groups also received a vitamin D supplement (calcifediol 266 *μ*g) at a dose of one vial orally every 15 days. This study found that levels of serum osteocalcin increased to a greater extent in the OHC group compared with the calcium carbonate group. Changes over time in serum osteocalcin level were also statistically significant (*P* < 0.05) in the OHC group, but not in the calcium carbonate group. Besides, changes in mean BMD at the lumbar spine and femoral neck between baseline and year 3 were −1.1% and 2.5% for OHC and −2.3% and 1.2% for calcium carbonate, respectively [20].

Previous studies have reported the application of microsized hydroxyapatite to bone regeneration, but the result is not satisfied. The limitation comes from the size of hydroxyapatite. In addition, the duration of treatment is very long. The recent development is applying the nanobiology approach to hydroxyapatite, although more studies are warranted especially its affinity. Nanobiology approach comes from the concept that the mineral atoms arranged in a crystal structure of hydroxyapatite can be substituted or incorporated by the other mineral atoms. A study by Noor et al. [13] who performed mineral atomic substitution modeling in bone hydroxyapatite crystals among Indonesian population showed changes in porosity and density of hydroxyapatite crystals in various mineral atomic substitutions. Some studies also applied substitution of magnesium [21], silicon [22], zinc [23], iron [24], fluoride [25], chloride [26], carbonate [27], and strontium [28] in hydroxyapatite crystals. The results demonstrated changes in the physicochemical properties and biological response of nanohydroxyapatite crystals. 

One benefit of nanocomposite is its ability to assemble with other molecules. These properties will increase the quality of osteoporosis management although need further study. In the present work, the bone regeneration potential of nanohydroxyapatite/chitosan composite scaffolds was compared with that of pure chitosan scaffolds when implanted into segmental bone defects in rabbits. Critical size bone defects (6 mm diameter, 10 mm length) were created in the left femoral condyles of 43 adult New Zealand white rabbits. The femoral condyle bone defects were repaired by nanohydroxyapatite/chitosan compositions, pure chitosan, or left empty separately. Defect-bridging was detected by plain radiograph and quantitative computer tomography at eight and 12 weeks after surgery. Tissue samples were collected for gross view and histological examination to determine the extent of new bone formation. Eight weeks after surgery, more irregular osteon formation was observed in the group treated with nanohydroxyapatite/chitosan composites compared with those treated with pure chitosan. Twelve weeks after surgery, complete healing of the segmental bone defect was observed in the nanohydroxyapatite/chitosan group, while the defect was still visible in the chitosan group, although the depth of the defect had diminished. These observations suggest that the injectable nanohydroxyapatite/chitosan scaffolds are potential candidate materials for regeneration of bone loss [29]. In addition, experiments have shown that one of the mechanisms of the contact action of nano-HAP on bacteria *Staphylococcus aureus *consists in adhesion of nanocrystals on bacteria with subsequent formation of a nanostructure of particles of reaction products hindering the enlargement and division of bacteria [30]. 

## 9. Conclusion

The basic elements of hydroxyapatite crystals composed of atomic minerals in certain geometric pattern and their relationship to bone cell biological activity have opened opportunities for hydroxyapatite crystals supplement application to osteoporosis. Understanding of the characteristics of bone hydroxyapatite crystals as well as the behavior of mineral atom in the substitution will have a better impact on the management of osteoporosis in the world.
